# Influence of Non-Thermal Atmospheric Pressure Plasma Treatment on Retentive Strength between Zirconia Crown and Titanium Implant Abutment

**DOI:** 10.3390/ma14092352

**Published:** 2021-05-01

**Authors:** Dae-Sung Kim, Jong-Ju Ahn, Gyoo-Cheon Kim, Chang-Mo Jeong, Jung-Bo Huh, So-Hyoun Lee

**Affiliations:** 1 Department of Prosthodontics, Dental Research Institute, Dental and Life Science Institute, School of Dentistry, Pusan National University, Busan 49, Korea; modesthanks@gmail.com (D.-S.K.); tarov0414@hanmail.net (J.-J.A.); cmjeong@pusan.ac.kr (C.-M.J.); neoplasia96@hanmail.net (J.-B.H.); 2 Department of Oral Anatomy and Cell Biology, School of Dentistry, Pusan National University, Busan 49, Korea; ki91000m@pusan.ac.kr; 3 Research & Development Center, FEAGLE Corporation, Busan 70-6, Korea

**Keywords:** non-thermal atmospheric pressure plasma, zirconia, titanium, crown, implant abutment, retentive strength, thermocycling, silane agent, MDP primer

## Abstract

The aim of this study is to investigate the effect of non-thermal atmospheric pressure plasma (NTP) on retentive strength (RS) between the zirconia crown and the titanium implant abutment using self-adhesive resin cement. Surface free energy (SFE) was calculated on 24 cube-shaped zirconia blocks, and RS was measured on 120 zirconia crown-titanium abutment assemblies bonded with G-CEM LinkAce. The groups were categorized according to the zirconia surface treatment as follows: Control (no surface treatment), NTP, Si (Silane), NTP + Si, Pr (Z-Prime Plus), and NTP + Pr. Half of the RS test assemblies were aged by thermocycling for 5000 cycles at 5–55 °C. The SFE was calculated using the Owens-Wendt method, and the RS was measured using a universal testing machine at the maximum load until failure. One-way analysis of variance (ANOVA) with post-hoc Tukey honestly significant difference (HSD) was performed to evaluate the effect of surface treatments on the SFE and RS. Independent sample t-test was used to compare the RS according to thermocycling (*p* < 0.05). For the SFE analysis, the NTP group had a significantly higher SFE value than the Control group (*p* < 0.05). For the RS test, in non-thermocycling, the NTP group showed a significantly higher RS value than the Control group (*p* < 0.05). However, in thermocycling, there was no significant difference between the Control and NTP groups (*p* > 0.05). In non-thermocycling, comparing with the NTP + Si or NTP + Pr group, there was no significant difference from the Si or Pr group, respectively (*p* > 0.05). Conversely, in thermocycling, the NTP + Si and NTP + Pr group had significantly lower RS than the Si and Pr group, respectively (*p* < 0.05). These results suggest that NTP single treatment for the zirconia crown increases the initial RS but has little effect on the long-term RS. Applied with Silane or Z-Prime Plus, NTP pre-treatment has no positive effect on the RS.

## 1. Introduction

In implant dentistry, zirconia is one of the most widely used prosthetic materials in recent years because of its excellent aesthetics, biological stability, and wear resistance [1,2,3]. Moreover, with the development of high-precision CAD-CAM systems, the processing method of zirconia has become simpler and more convenient than that of metal-ceramic when manufacturing implant prostheses [4]. According to a systematic review, the five-year survival rate for implant-supported single crowns was 98.3% for metal-ceramic and 97.6% for zirconia with no statistically significant differences between them [5].

Despite this high survival rate, loss of retention is one of the most frequent technical complications for overall implant prostheses, including zirconia implants prosthesis [5,6]. Based on the previous study, the rate of technical complications was 2–9% for veneer fractures, 3–8% for abutment/screw loosening, and 5% for retention loss over the approximately five-year follow-up period [6]. In particular, when bonding zirconia with resin cement, conventional ceramic bonding techniques provide insufficient bond strength [7]. For feldspathic ceramic, the bonding strength with resin cement increases through micromechanical retention with hydrofluoric acid and chemical bonding using a silane agent [8,9]. However, these methods are ineffective for zirconia because of its polycrystalline structure that contains little silica and forms high corrosion resistance [10,11,12]. To overcome this limitation and increase the RS of zirconia implant prosthesis, various surface treatment methods, such as sandblasting, tribochemical silica coating, and application of primer including functional monomer have been introduced [13,14].

Sandblasting is a traditional way to promote bond strength through micromechanical interlocking between zirconia and resin cement [15,16,17,18]. However, sandblasting can cause microcracks on the zirconia surface, which leads to a fracture or damage on the zirconia crown margin [9]. Tribochemical silica coating converts the zirconia surface from the silica-free state to the silica-rich state and enables siloxane bonds by silanization [19]. Nevertheless, the previous study has shown that this technique has no significant benefit and only provides the effect of surface roughness similar to sandblasting with aluminum oxide [20]. 10-Methacryloyloxydecyl dihydrogen phosphate (MDP) application on the zirconia surface provided a zirconium-oxygen-phosphorus bond (Zr-O-P) like the siloxane bond (Si-O-Si) formed by treating the tribochemical silica coating and silane agent [21,22]. However, some previous studies have reported that applying an MDP primer to sintered zirconia did not maintain stable bond strength after thermocycling [23,24]. Given the above advantages and drawbacks, no conclusive surface treatment has existed for efficient bonding to zirconia prosthesis [25,26,27]. Therefore, there is a need to research alternative methods for biologically safe and long-lasting zirconia bonding.

Plasma surface treatment is a method for modifying the surface of materials and has long been used in various industrial and medical fields [28]. Highly reactive species such as electrons, free radicals, ions, and electronically excited neutrons are found in plasma [29,30]. These plasma species improve surface reactivity with little change in the intrinsic properties of the materials [31,32]. In particular, among plasma types, non-thermal atmospheric pressure plasma (NTP) does not require the vacuum equipment necessary for vacuum plasma [31]. In addition, NTP generation equipment is not only inexpensive but also low in maintenance costs [31]. This NTP processing has been introduced to dentistry and may be an effective tool for increasing the RS of the zirconia crown [33,34].

Several articles have reported the effect of NTP on shear bond strength or microtensile bond strength between zirconia and resin cement [32,33,34,35,36,37,38]. However, there is currently no study on the RS between NTP-treated zirconia crown and titanium implant abutment through resin cement. Moreover, the research comparing the RS by applying a silane agent or an MDP primer after NTP pre-treatment to the zirconia crown has not been reported so far. Accordingly, the purpose of this study is to evaluate the effect of NTP as a surface treatment of the zirconia crown for bonding to the titanium implant abutment via the resin cement. This research also compared the effects of each on the RS by treating either the silane agent or the MDP primer after NTP pre-treatment. The first null hypothesis was that NTP single treatment does not increase the RS between the zirconia crown and the titanium implant abutment regardless of thermocycling. The second null hypothesis was that NTP pre-treatment does not increase the RS when applied with the silane agent or the MDP primer regardless of thermocycling.

## 2. Materials and Methods

### 2.1. Preparation of Zirconia Specimens and Titanium Implant Abutments

**Figure 1:**
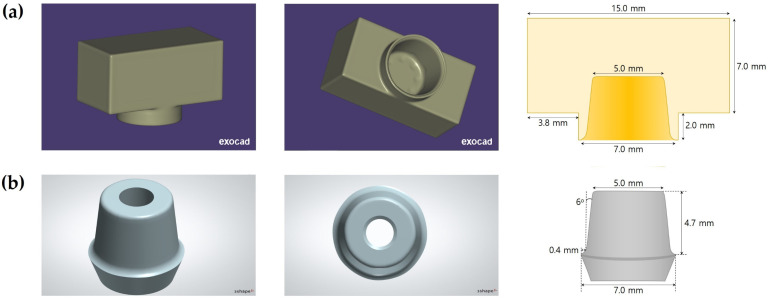
(**a**) CAD design of the zirconia crown and schematic diagram of the cross-section of the zirconia crown. (**b**) CAD design and schematic diagram of the titanium abutment.

**Figure 2:**
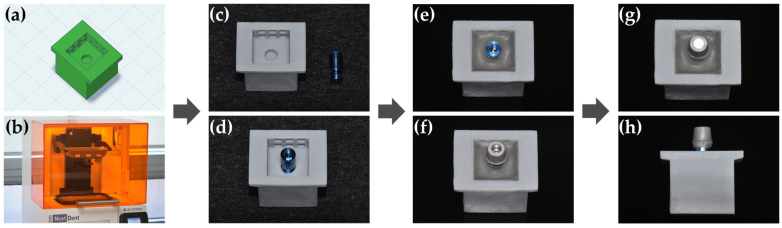
A process of lab analog embedding and titanium abutment fixation. (**a**) CAD data of the jig. (**b**) 3D printing process. (**c**) 3D-printed jig and lab analog. (**d**) Lab analog in the jig hole. (**e**) Lab analog embedded in acrylic resin. (**f**) Titanium abutment fixed to a lab analog with 35 Ncm torque. (**g**) Filling screw hole using Teflon tape. (**h**) Complete lab analog-titanium abutment connection.

The 24 cube-shaped zirconia blocks (10 × 10 × 5 mm^3^) were prepared for surface free energy and XPS analysis, and the 120 zirconia crowns were prepared for the RS test. For the zirconia crown, a cement gap of 100 μm was provided for bonding with the titanium abutment using a CAD program (Exocad, Exocad GmbH, Darmstadt, Germany). The diameter of the zirconia crown is 7.0 mm, and the height is 9.0 mm. In addition, areas for hanging wire were assigned to the upper left and right sides of the crown for the RS test (Figure 1). The manufacturing process was performed by milling pre-sintered zirconia blocks (LUXEN, DentalMax, Seoul, Korea) and sintering them according to the manufacturer’s instructions. The 120 titanium abutments were designed with a taper of 6° in the axial wall, 7.0 mm in the diameter of the margin area, and 4.7 mm in height using CAD software (3Shape’s CAD design software, 3Shape, Copenhagen K, Denmark) (Figure 1). Each abutment was manufactured by milling a titanium pre-milled bar with an external hexagonal connection (CEHE011, DoowonID Co., Ltd., Daejeon, Korea), according to the manufacturer’s instructions. For the RS test, a jig was fabricated using CAD software (Tinkercad, Autodesk Inc., San Francisco, CA, USA) and a 3D printer (NextDent 5100, 3D Systems, Inc., Rock Hill, SC, USA). A laboratory analog (US Fixture Lab analog, Osstem Implant, Busan, Korea) was fixed to the jig, embedded with acrylic resin, connected to the titanium abutment, and a torque of 35 Ncm was applied. A screw hole of the titanium abutment was filled with Teflon tape (Figure 2).

**Figure 3:**
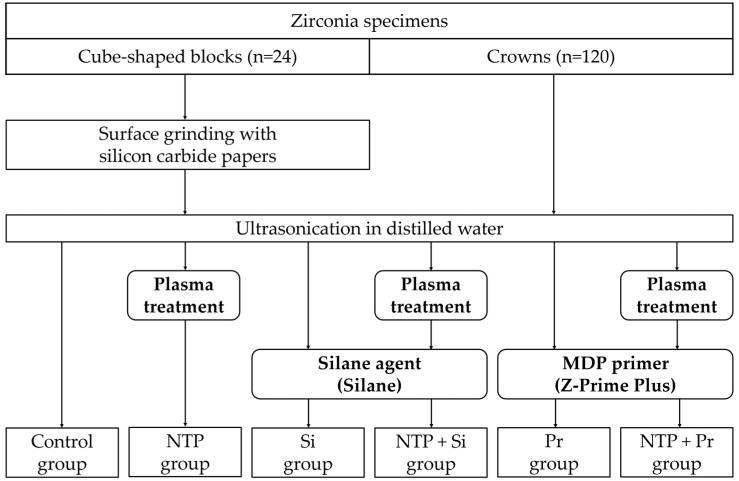
Flowchart of the surface treatment process for zirconia specimens.

The surface treatments for the zirconia specimens are the following: no surface treatment (Control), non-thermal atmospheric pressure plasma (NTP), Silane (Si), NTP followed by Silane (NTP + Si), Z-Prime Plus (Pr), and NTP followed by Z-Prime Plus (NTP + Pr). The sandblasting was conducted vertically on the titanium abutment with a dental sandblaster (Basic master, Renfert, Hilzingen, Germany) at a pressure of 2.5 bar for 15 s. This procedure was carried out at a distance of 10 mm from the titanium abutment surface using 50 μm of aluminum oxide (Al_2_O_3_) particles (Hi-Aluminas, Shofu Inc., Kyoto, Japan). The cube-shaped zirconia blocks were polished for 60 s at 300 rpm by attaching 600 grit and 800 grit paper to the grinding machine (MetaServ 250, Buehler, Lake Bluff, IL, USA). All cube-shaped zirconia blocks, zirconia crowns, and titanium abutments were washed with distilled water in an ultrasonic cleaner (SH-1025, Saehan ultrasonic Co., Ltd., Seoul, Korea) for 3 min and then dried using an air syringe. The whole procedure for the surface treatments of zirconia specimens is summarized in Figure 3. Table 1 shows the number of zirconia specimens distributed across the entire experimental group.

### 2.2. Non-Thermal Atmospheric Pressure Plasma (NTP) Treatment

**Figure 4:**
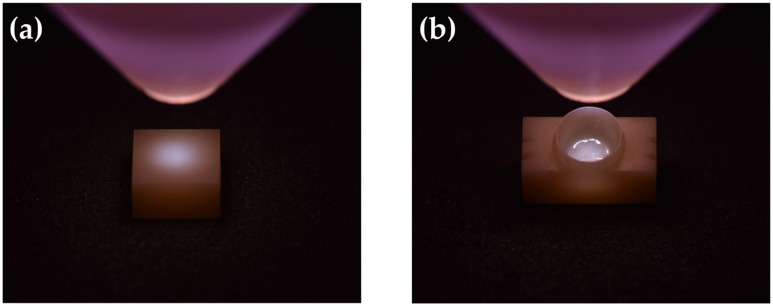
(**a**) Non-thermal atmospheric pressure plasma (NTP) treatment on the cube-shaped zirconia block. (**b**) NTP treatment on the inner surface of the zirconia crown.

NTP treatment was performed on 12 cube-shaped zirconia blocks and 60 zirconia crowns using a plasma generating device (FG-Explorer, FEAGLE, Yangsan, Korea). Argon was used as the working gas with the flow rate of 5.0 slm (standard liter per minute), the peak plasma discharge current was 4.80 mA, and the peak-to-peak applied voltage was 3 kVpp. NTP generated by this device forms the plasma plume inside the plasma source and does not extend the plume outwardly like a plasma jet. The plasma source tip was placed 10 mm above the surface of the cube-shaped zirconia block and the inner occlusal surface of the zirconia crown. The tip was positioned perpendicularly to the zirconia surface so that NTP was evenly applied for 1 min (Figure 4) [35].

### 2.3. Contact Angle Measurement and Surface Free Energy (SFE) Analysis

Of the 24 cube-shaped zirconia blocks, 18 were divided into three per surface treatment group. The contact angle was investigated using two different liquids: water as the polar liquid and diiodomethane as the dispersive liquid. Each liquid was applied with a sessile drop technique once per zirconia block. The contact angle of each liquid was measured as the average value of the right and left sides using a computer-controlled image analyzer equipped with a video camera (Ramé-Hart 190-U1, Ramé-Hart Instrument Co., Succasunna, NJ, USA). The SFE for each group was calculated based on the contact angle value using the Owens-Wendt method [35].

### 2.4. X-ray Photoelectron Spectroscopy (XPS) Analysis

To evaluate the chemical composition of the zirconia surface, X-ray photoelectron spectroscopy (XPS) (AXIS SUPRA, Kratos Analytical Ltd., UK) analysis was performed before and after NTP treatment. Of the 24 cube-shaped zirconia blocks, six were assigned per surface treatment group. The blocks were placed on carbon tape, and XPS data were acquired using a monochromatic Al-Kα X-ray source (1486.6 eV) at 15 kV and 225 W. All composition measurements were acquired at the surface normal with charge neutralization. The binding energy scale was calibrated using a C1s level of 284.5 eV. The angle between the X-ray source and the analyzer was 54.7°. A 165 mm mean radius hemispherical sector analyzer was used as an electron energy analyzer, operating in fixed analyzer transmission (FAT) mode. For each block, a compositional survey scan was acquired using a pass energy of 160 eV and core level spectra with a pass energy of 20 eV. The block was placed in a vacuum chamber of 5 × 10^−9^ Torr. Data analysis was performed using data reduction software (Vision 1.5, Kratos Analytical Ltd., Kratos Analytical Ltd., Manchester, UK). The deconvoluted spectra were fitted with a Gaussian−Lorentzian sum function (20% Gaussian and 80% Lorentzian) using the XPS peak fitting program provided by Raymund WM Kwok (XPS PEAK 4.1, The Chinese University of Hongkong, Shatin, Hong Kong).

### 2.5. Retentive Strength (RS) Test before and after Thermocycling

**Figure 5:**
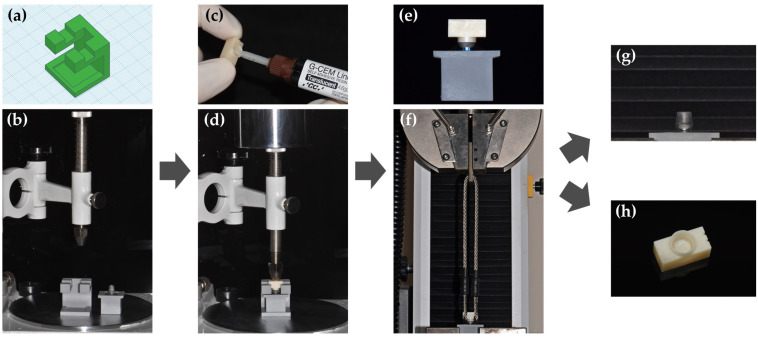
The process of testing retentive strength (RS) between the zirconia crown and the titanium abutment. (**a**) CAD data of the positioning stand. (**b**) 3D-printed positioning stand and titanium abutment on a surveyor. (**c**) Application of G-CEM LinkAce inside the zirconia crown. (**d**) Zirconia crown and titanium abutment bonded with the resin cement by a force of 50N using a surveyor. (**e**) Zirconia crown-titanium abutment assembly after light polymerization. (**f**) Fixing the assembly to the Instron machine and placing the wire on the zirconia crown. (**g**) Debonded titanium abutment after the RS test. (**h**) Debonded zirconia crown after the RS test.

All the zirconia crowns were distributed 20 per surface treatment group. G-CEM LinkAce (GC Corporation, Tokyo, Japan), a self-adhesive resin cement, was used for the RS test. Depending on the method of surface treatment defined for each group, the silane agent or the MDP primer was used. The information of the cement, silane agent, and MDP primer is shown in Table 2. Silane agent (Silane, Ultradent Products Inc., South Jordan, UT, USA), containing 3-methacryloxypropyltrimethoxysilane (MPS), was uniformly applied to the inner surface of the crown using a microbrush and left to dry for 60 s. The MDP primer (Z-Prime Plus, Bisco Inc., Schaumberg, IL, USA), containing 10-MDP, was evenly applied to the inside of the crown using a microbrush and dried with compressed air for 5 s. The cement was injected into the crown, and then the crown was seated with a constant force of 50 N on the titanium abutment using a dental surveyor (SURVEYOR-II, SAESHIN Precision Co., Ltd., Daegu, Korea). Excess cement in the margin area was removed using the microbrush. The cement was then light polymerized in three directions with an LED curing light (Elipar™ DeepCure-L, 3M ESPE, St. Paul, MN, USA) at 1000–1200 mW/cm^2^ for 20 s. In this process, a customized positioning stand made by a 3D printer was used to ensure that the wire was hung in the same direction on each crown during the RS test (Figure 5).

Before the RS test, half of the zirconia crown-titanium abutment assemblies were stored in distilled water for 24 h at 37 °C after the polymerization. The rest went through thermocycling with a thermocycling machine (TW-P01, Taewontech, Bucheon, Korea) for 5000 cycles at 5–55 °C, 2 s interval between the baths, and 30 s per temperature. The RS was then measured at the maximum load up to failure at a 1.0 mm/min crosshead speed using a universal testing machine (Instron 3345, Instron, Norwood, MA, USA) (Figure 5).

### 2.6. Failure Mode Analysis

Following the RS test, the surfaces of zirconia crowns and titanium abutments were examined at a magnification of 40× using an optical microscope (BX51, Olympus, Tokyo, Japan). Failure modes were categorized as follows: Adhesive failure between the zirconia crown and the resin cement or between the titanium abutment and the resin cement, cohesive failure in the resin cement, and mixed failure where adhesive and cohesive failures occurred simultaneously. Representative surfaces of debonded crowns and titanium abutments were plated with platinum (E1010, HITACHI, Tokyo, Japan) and evaluated with FE-SEM (S-4700, HITACHI, Tokyo, Japan). Before this analysis, zirconia crowns were embedded in acrylic resin and cross-sectioned with a low-speed diamond saw machine (Diamond Saw, SPG Co., Ltd., Incheon, Korea). 

### 2.7. Energy-Dispersive X-ray Spectroscopy (EDS) Analysis

For the energy-dispersive X-ray spectrometer (EDS) analysis, an additional zirconia crown-titanium abutment assembly corresponding to the experimental conditions of the NTP group was fabricated. After thermocycling, the assembly was embedded in acrylic resin and cross-sectioned with a low-speed diamond saw machine (Diamond Saw, SPG Co., Ltd., Incheon, Korea). The EDS (Quantax XFlash 6|60 detector, Bruker Nano GmbH, Berlin, Germany) connected to the FE-SEM was used to observe the chemical element distribution at the cross-sectional bonded interface. Line scan analysis and elemental mapping analysis were performed during EDS analysis.

### 2.8. Statistical Analysis

One-way analysis of variance (ANOVA) was conducted to evaluate the effect of surface treatments on the SFE and RS, and post-hoc tests with Tukey honestly significant difference (HSD) were used. The RS values according to thermocycling were compared using the independent sample t-test (*p* < 0.05). A software program (SPSS Statistics V24, IBM Corp., Chicago, IL, USA) was used to perform all statistical analyses.

## 3. Results

### 3.1. Contact Angle Measurement and Surface Free Energy (SFE) Analysis

**Figure 6:**
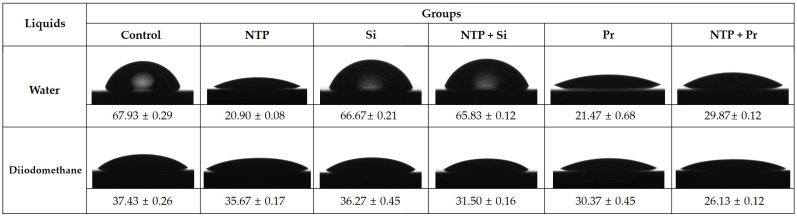
Contact angle images and values of water and diiodomethane droplets on zirconia surfaces. Control, no treatment. NTP, non-thermal atmospheric pressure plasma. Si, Silane. NTP + Si, NTP followed by Silane. Pr, Z-Prime Plus. NTP + Pr, NTP followed by Z-Prime Plus.

**Figure 7:**
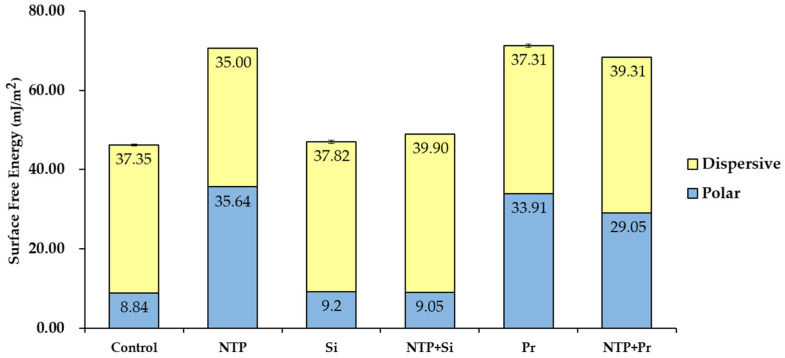
Average polar and dispersive values of SFE for zirconia surface. The sum of both components represents the total SFE value.

Representative contact angle images and values of each group for SFE analysis are shown in Figure 6. The mean values of the polar and dispersive portions of SFE are also illustrated in Figure 7, and the sum of these values is the total values of SFE for each group. The differences in the SFE value according to the surface treatment were mainly due to the change of the polar portion rather than the dispersive portion of SFE. Among the groups not treated with NTP, the Pr group had a significantly higher SFE value than the Control and Si groups (*p* < 0.05). Among the NTP treatment groups, the NTP group had a significantly higher SFE value than the NTP + Si and NTP + Pr groups (*p* < 0.05). In addition, the NTP + Pr group had a significantly higher SFE value than the NTP + Si group (*p* < 0.05). Comparing the effect of NTP on SFE, the NTP group had a significantly higher SFE value than the Control group (*p* < 0.05). In addition, the NTP + Si group had a significantly higher SFE value than the Si group (*p* < 0.05). Conversely, the NTP + Pr group had a significantly lower SFE value than the Pr group (*p* < 0.05).

### 3.2. X-ray Photoelectron Spectroscopy (XPS) Analysis

**Figure 8:**
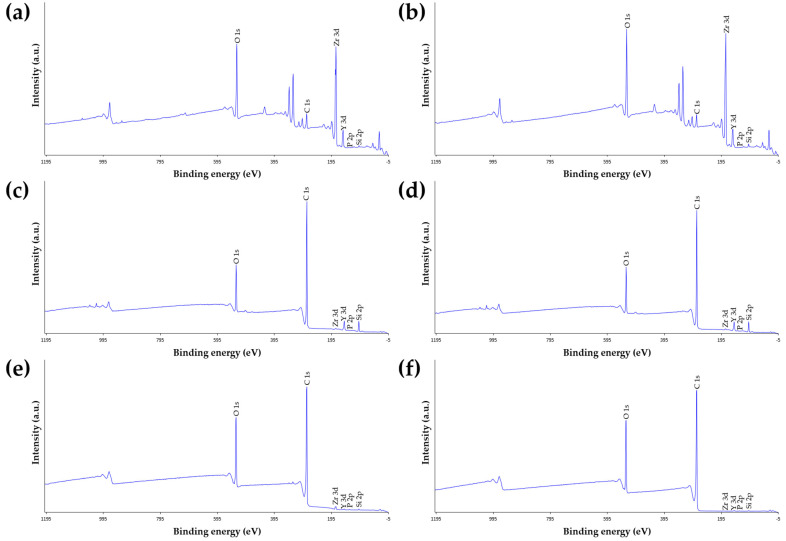
Survey XPS spectra for the zirconia blocks before and after NTP treatment. (**a**) Control group. (**b**) NTP group. (**c**) Si group. (**d**) NTP + Si group. (**e**) Pr group. (**f**) NTP + Pr group.

A representative XPS spectrum of the zirconia surface before and after plasma treatment is shown in Figure 8. Surface chemistry composition for all six groups before and after plasma treatment are shown in Table 3. Compared to the Control group, the atomic percentage of carbon (C) decreased but that of oxygen (O) increased in the NTP group. On the other hand, compared to the Si and Pr groups, the atomic percentage of carbon increased but that of oxygen decreased in the NTP + Si and NTP + Pr groups, respectively.

### 3.3. Retentive Strength (RS) Test

**Figure 9:**
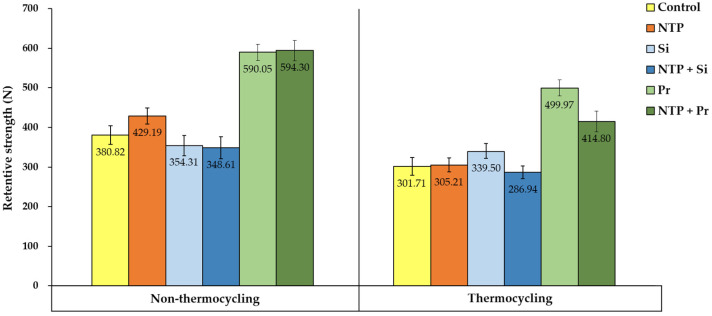
Average RS values between the zirconia crown and the titanium abutment according to thermocycling and surface treatment.

The RS values for each group according to thermocycling and surface treatments are shown in Figure 9. First, the different surface treatment effects were evaluated by comparing the RS values for the same thermal effect. Regardless of thermocycling, among the groups not treated with plasma, the Pr group had a significantly higher RS value than the Control and Si groups (*p* < 0.05). Among the NTP treatment groups, the NTP + Pr group had a significantly higher RS value than the NTP and NTP + Si groups (*p* < 0.05). Comparing the effect of NTP treatment, in non-thermocycling, the NTP group had a significantly higher RS value than the Control group (*p* < 0.05). However, the RS values of the Si and Pr groups were not significantly different from the NTP + Si and NTP + Pr groups, respectively. In thermocycling, there was no significant difference between the Control and NTP groups. On the other hand, the Si and Pr groups had significantly higher RS values than the NTP + Si and NTP + Pr groups, respectively (*p* < 0.05).

Second, the RS values were compared to determine the thermal effect for the same surface treatment. Regardless of surface treatment, all the groups had significantly lower RS values in thermocycling than in non-thermocycling (*p* < 0.05).

### 3.4. Failure Mode Analysis

**Figure 10:**
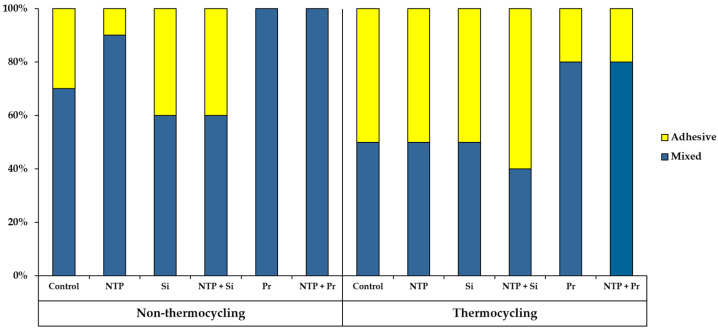
Failure mode between the zirconia crown and the resin cement in all groups after RS test. Adhesive failure between the titanium abutment and the resin cement, as well as cohesive failure in the resin cement, did not occur in any group.

**Figure 11:**
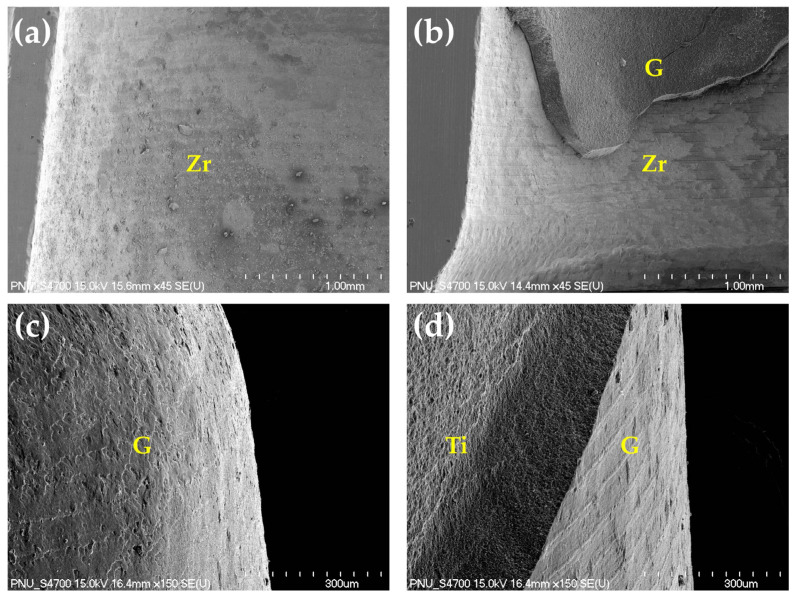
(**a**,**b**) Representative FE-SEM images (45×) of the inner surface for zirconia crown in thermocycling after the RS test. (**a**) Adhesive failure of the Control group. (**b**) Mixed failure of the NTP group. (**c**,**d**) Representative FE-SEM images (150×) of the titanium abutment surface in thermocycling after the RS test. (**c**) The surface where adhesive failure between the zirconia crown and the resin cement occurred in the Control group. The entire surface of the titanium abutment was covered with the resin cement. (**d**) The surface where the mixed failure occurred in the NTP group. Zr, zirconia surface. G, G-CEM LinkAce. Ti, titanium abutment.

Figure 10 shows the pattern of failure modes for all test groups as a percentage. Adhesive failure between the titanium abutment and resin cement, as well as cohesive failure in the resin cement, did not appear in any group. Compared with non-thermocycling, the percentage of adhesive failure between the zirconia crown and the resin cement increased for all the groups in thermocycling. In non-thermocycling, the percentage of adhesive failure between the zirconia crown and the resin cement was higher in the Control group than in the NTP group. Figure 11 shows representative FE-SEM images of the zirconia crown and the titanium abutment surface when the adhesive and mixed failures occurred for the Control and NTP groups in thermocycling.

### 3.5. Energy-Dispersive X-ray Spectroscopy (EDS) Analysis

**Figure 12:**
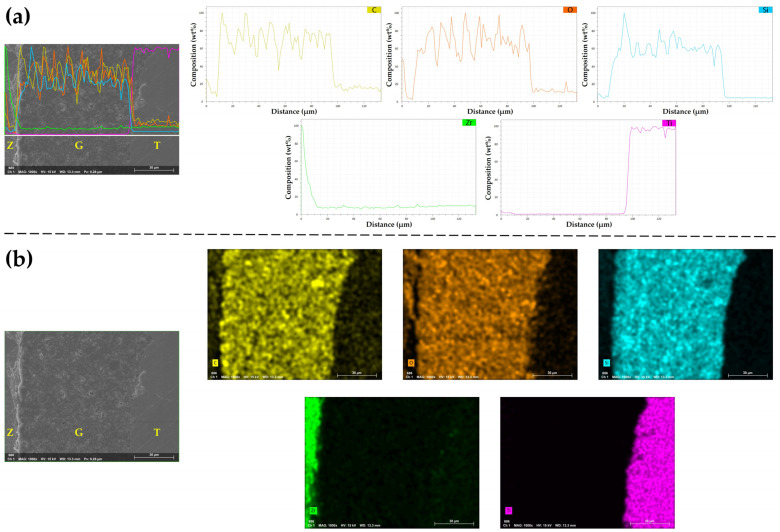
Line scan analysis and elemental mapping analysis at the same cross-sectional bonding interface for the NTP group in thermocycling. (**a**) Line scan analysis shows the distribution of each element along the white straight line marked in the FE-SEM image. (**b**) Elemental mapping analysis shows the presence of each element through color differences. Z, Zirconia surface. G, G-CEM LinkAce. T, Titanium abutment. C, Carbon. O, Oxygen. Si, Silicon. Zr, Zirconium. Ti, Titanium.

For an additional zirconia crown-titanium abutment assembly treated with NTP and thermocycling, line scan analysis was performed using the EDS to determine the elemental distribution of the cross-sectional bonding interface. Among the region of straight lines selected on the FE-SEM image, large changes in the ion distribution of Zr, C, O, and Si were observed at the bonding interface between the zirconia crown and the resin cement. The thickness of the ion distribution change zone was approximately 3–4 μm. The element distribution of the cross-section was observed through elemental mapping analysis, and each color point represents the distribution of each element at a specific part of the FE-SEM image. Elemental mapping analysis showed the elements identified in the line scan analysis, and the distribution of C, O, and Si at the bonding interface was also observed (Figure 12).

## 4. Discussion

Non-thermal atmospheric pressure plasma (NTP) has recently been introduced to replace conventional mechanical surface treatment to improve the bond strength of zirconia [37]. Unlike sandblasting and tribochemical silica coating, NTP uses an ionizing inert gas to treat the zirconia surface at an electronic level. Thus, NTP has little effect on the surface roughness and can reduce the risk of microcracks [10,39,40,41]. In addition, since NTP does not use Al_2_O_3_ particles, there is no need to go through the cleaning process after the surface treatment [35]. In particular, the argon gas used in this experiment is widely used for NTP due to its high cleaning effect, low ionization energy, and economical cost [28,31,42]. In this study, the SFE, XPS, and EDS were measured, respectively, to investigate the effect of NTP treatment on the zirconia surface.

Several studies have reported that applying NTP improves the wettability and SFE of zirconia [38,40,41]. NTP processing breaks down the moisture in the plasma gas and atmosphere with high-energy electrons to generate OH radicals [33,42,43]. These radicals eliminate organic pollutants on the zirconia surface by breaking the C-C and C-H bonds [33,42,43]. This is consistent with the XPS analysis of our study. XPS analysis showed that the carbon percentage of the NTP group was lower than that of the Control group. Accordingly, it suggests that surface organic impurities containing carbon were removed by NTP treatment. The O/C ratio as an indicator of surface wettability has been generally used to specify the number of functional groups containing oxygen on the plasma-treated surface [44,45,46]. High O/C ratios indicate good wettability of the surface [39]. XPS analysis showed a higher oxygen percentage in the NTP group than in the Control group. The decrease of the carbon percentage and increase of the oxygen percentage due to NTP treatment showed a high O/C ratio. These results can explain why NTP treatment reduces the contact angle of zirconia and increases the SFE in this study.

EDS conducted in the current study examined the cross-section of the zirconia-titanium abutment assembly treated with NTP and thermocycling. This procedure was performed to determine if chemical bonds were formed between NTP-treated zirconia crown and self-adhesive resin cement. According to the manufacturer, G-CEM LinkAce, which is a self-adhesive resin cement used in the experiment, contains SiO_2_ and phosphoric acid ester monomer [35]. As a result of the elemental mapping analysis, it was confirmed that the main components of the resin cement were carbon (C), oxygen (O), and silicon (Si). C, O, and Si were hardly observed at the zirconia except for the boundary region. Zirconium (Zr) was hardly detected in the resin cement except for the boundary region. In line scan analysis, each element distribution was obtained by analyzing the spectral lines over distance. Line scan analysis revealed C, O, Si, and Zr elements at the interface between the zirconia and the resin cement. Although further research is needed, these results indicate that a stable bond might be formed between the zirconia and the resin cement, taking into account the RS values observed in the NTP group with thermocycling [47].

In our study, we applied G-CEM LinkAce after NTP treatment on the inner surface of the zirconia crown and performed the RS test between the zirconia crown and the titanium abutment. For this experiment, sandblasting was not performed on the inner surface of the zirconia crown to see only the chemical effects of the surface treatments. In non-thermocycling, the RS value of the Control group was significantly lower than that of the NTP group. Through the XPS analysis, as compared to the Control group, the atomic percentage of oxygen increased in the NTP group. This result indicates that NTP treatment increased the oxygen content on the zirconia surface. As a result, more intermolecular secondary forces, such as van der Waals bonds, might occur between the hydroxyl groups on the zirconia surface and the resin cement [30,38]. However, in thermocycling, no significant differences were found between the Control and NTP groups. These findings correspond to previous studies where the bond strength of NTP-treated zirconia decreased significantly after thermocycling [32,34,48]. Thus, the RS may be associated with a low resistance to thermal stress and hydrolysis of chemical bonds following NTP processing [49,50]. In particular, previous studies have reported that, when resin cement comes in contact with oxygen, oxygen reacts with radical polymerization catalysts to stimulate resin monomer molecules and prevent them from binding to each other [51,52]. Consequently, the immediate increase in the oxygen level of the zirconia surface due to NTP treatment may be an obstacle to the polymerization of self-adhesive resin cement. Taken together, we propose that NTP processing can help increase the initial RS between the zirconia crown and the titanium abutment. The high initial RS can prevent zirconia prosthesis from being prematurely debonded [35].

The effect of the combination of two surface treatments was examined: Silane or Z-Prime Plus after NTP treatment. According to several articles, sandblasting and MDP primer have been widely used for zirconia surface treatment [23,25,53]. In addition, tribochemical silica coating is performed by treating silica-coated Al_2_O_3_ particles and then applying a silane agent [19]. However, in this study, the two methods that could affect the surface roughness were excluded. Instead, NTP that hardly damages the surface was pretreated to investigate the effect on the RS. When selecting the silane agent and the MDP primer as the experimental material, the products containing both 3-MPS and 10-MDP were excluded to determine the NTP effect for the single component. Silane, which is a silane agent, has been reported to contain more 3-MPS than other products, which is convenient for use as a syringe-type [53]. Z-Prime Plus, which is an MDP primer, has been reported to improve bond strength over other products in previous studies [12,54].

In terms of the RS, the effects of Silane or Z-Prime Plus on the NTP pre-treated zirconia crown were compared in the study. Regardless of thermocycling, the RS value of the Pr group was significantly higher than the Si group. Furthermore, the NTP + Pr group had a significantly higher RS value than the NTP + Si group. According to previous reports, using Silane alone is not effective on stable zirconia surfaces [53,55,56]. Other studies have also found that the longer the carbon chain length of the monomer, the better the bonding effect [12,57,58]. 10-MDP of Z-Prime Plus used in the experiment has more carbon chains than 3-MPS of Silane, which may affect the RS test results [21,59]. Moreover, P-OH of 10-MDP is thought to respond better to the NTP pre-treated zirconia surface than Si-OH of 3-MPS.

The combination of NTP and Silane and that of NTP and Z-Prime Plus were also compared with the single treatment of Silane and that of Z-Prime Plus, respectively. In non-thermocycling, there were no significant differences on the RS between the Si and NTP + Si groups as well as the Pr and NTP + Pr groups. Regarding our results, the previous study has reported that NTP has a limited effect on the reaction with materials with high surface free energy and ionic components [60]. In addition, Balkenhol et al. [61] have reported no significant increase in bond strength between zirconia and resin cement when the MDP primer was used with NTP treatment. Our XPS analysis may also support the result. The difference in oxygen percentage between the Pr and the NTP + Pr groups, as well as the Si and the NTP + Si groups, was smaller than between the Control and NTP groups. In thermocycling, the NTP + Si and NTP + Pr groups had significantly lower RS values than the Si and Pr groups, respectively. In other words, NTP treatment before applying Silane or Z-Prime Plus to the zirconia crown may have a negative effect on the RS under thermal stress.

As a result of the failure mode analysis after the RS test, in non-thermocycling, the adhesive failure rate between the zirconia crown and the resin cement was lower in the NTP group than in the Control group. However, in thermocycling, the percentage of adhesive failure for the NTP group was the same as the Control group. It is thought that the NTP treatment may increase the hydrophilicity of the zirconia surface, accelerating the hydrolysis at the adhesive interface. Therefore, it is considered that an additional process should be considered to increase the hydrolysis resistance at the bonding interface during NTP treatment. On the other hand, no adhesive failure between the titanium abutment and the resin cement occurred in any group in our experiment. For our research, titanium round bars were milled with CAD-CAM to process abutments of smooth surfaces, followed by sandblasting. Generally, sandblasting increases the surface area and surface roughness [62]. In addition, sandblasted rough titanium abutment surfaces provide higher micromechanical retention than smooth surfaces [63]. It is thought that there was no adhesive failure due to the increased mechanical bond between titanium abutment and resin cement.

To perform the RS test, the jig and stand for the assemblies were made using 3D printing. In the initial design of the study protocol, the RS test was performed with the cylinder-shaped lab analog fixed to the Instron machine. As a result, the slippage of the lab analog occurred, and it was difficult to determine whether the long axis of the zirconia crown was perpendicular to the floor. Additionally, the position of the crown relative to the wire was not constant, which caused the crown to be torqued when the wire was pulled. To minimize these errors, we created the 3D-printed jig that can embed the lab analog. The wings were also designed on the jig so that the long axis of the zirconia crown-titanium abutment assembly was perpendicular to the floor. Moreover, the 3D-printed positioning stand was used to keep the direction of the area for hanging wire constant when bonding the crown to the abutment. Through this process, the wire was hung adequately on the zirconia crown during the RS test. Proper wire hanging prevented the twisting of the wire connected to the Instron and the torque generation.

One of the limitations in the current study is that the period of thermocycling to water saturation was insufficient to assess hydrolysis durability [12,17]. In further studies, it is necessary to investigate the effect of thermal aging according to various surface treatments through long-term water storage. We did not determine the effects of mechanical loading on the RS. Future works should evaluate the impact of chewing simulation on the RS between the zirconia crown and the titanium abutment. Furthermore, NTP treatment was only performed on the inner surface of the zirconia crown. In future research, NTP processing on titanium abutment should be investigated for its effect on the RS.

## 5. Conclusions

Within the limitations of the current study, the following conclusions are made according to the zirconia surface treatments.

- NTP single treatment increases SFE of zirconia.
- NTP single treatment increases the initial RS between zirconia crown and titanium implant abutment but has little effect on the RS after thermocycling.
- Regardless of thermocycling, NTP pre-treatment does not show a positive effect on the RS when applied with Silane or Z-Prime Plus.
- Regardless of NTP pre-treatment and thermocycling, Z-Prime Plus shows higher RS than Silane.

## Figures and Tables

**Table 1:** Distribution of zirconia specimens for the entire experimental group. Control, no treatment. NTP, non-thermal atmospheric pressure plasma. Si, Silane. NTP + Si, NTP followed by Silane. Pr, Z-Prime Plus. NTP + Pr, NTP followed by Z-Prime Plus.

Groups	Distribution of Zirconia Specimens (n)			
	Cube-Shaped Blocks		Crowns	
	Surface Free Energy (SFE) Analysis	X-ray Photoelectron Spectroscopy (XPS) Analysis	Retentive Strength (RS) Test	
			Non-Thermocycling	Thermocycling
Control	3	1	10	10
NTP	3	1	10	10
Si	3	1	10	10
NTP + Si	3	1	10	10
Pr	3	1	10	10
NTP + Pr	3	1	10	10

**Table 2:** Material properties used in retentive strength (RS) test.

Material	Manufacturer	Type	Composition
G-CEM LinkAce	GC Corporation, Tokyo, Japan	Self-adhesive resin cement	Paste A: fluoroalumino silicate glass, urethane dimethacrylate (UDMA), dimethacrylate, pigment, silicon dioxide, initiator, inhibitor
			Paste B: urethane dimethacrylate (UDMA), dimethacrylate, phosphoric acid ester monomer, initiator, stabilizer
Silane	Ultradent Products Inc., South Jordan, UT, USA	Silane agent	3-Methacryloxypropyltrimethoxysilane (MPS), isopropyl alcohol
Z-Prime Plus	Bisco Inc., Schaumberg, IL, USA	MDP primer	Ethanol, bisphenol A-glycidyl methacrylate (Bis-GMA), 2-hydroxyethyl methacrylate, 10-methacryloyloxydecyl dihydrogen phosphate (MDP)

**Table 3:** Surface chemistry **composition** of zirconia blocks (in atomic percentage) for each group.

Elements	Groups					
	Control	NTP	Si	NTP + Si	Pr	NTP + Pr
C	21.10	14.56	71.14	79.33	81.19	82.72
O	49.90	55.96	16.97	12.02	17.61	16.77
Zr	20.25	20.76	0.17	0.15	0.37	0.00
Si	1.42	2.45	9.92	7.00	0.52	0.33
P	0.02	0.02	0.12	0.21	0.23	0.10
Y	7.25	6.24	1.39	1.07	0.07	0.06

## Data Availability

Not applicable.
